# Atoms in Generalized Orbital Configurations: Towards Atom-Dedicated Density Functionals

**DOI:** 10.3390/ijms20235943

**Published:** 2019-11-26

**Authors:** Ana Maria Toader, Maria Cristina Buta, Dan Maftei, Mihai V. Putz, Fanica Cimpoesu

**Affiliations:** 1 Institute of Physical Chemistry, Splaiul Independentei 202, 060021 Bucharest, Romania; atoader@icf.ro (A.M.T.); butamariacristina@gmail.com (M.C.B.); 2 Department of Chemistry, Universitatea Alexandru Ioan Cuza, 700506 Iaşi, Romania; dan.maftei@chem.uaic.ro; 3 Laboratory of Computational and Structural Physical-Chemistry for Nanosciences and QSAR, Biology-Chemistry Department, Faculty of Chemistry, Biology, Geography, West University of Timișoara, RO-300115 Timișoara, Romania; 4 Laboratory of Renewable Energies-Photovoltaics, R&D National Institute for Electrochemistry and Condensed Matter, RO-300569 Timișoara, Romania

**Keywords:** atomic electronic structure, Slater–Condon parameters, exchange energy, density functional theory (DFT), atomic basis sets

## Abstract

Deriving a practical formula for the atomic body with generalized shell occupations, we perform a detective analysis of the radial distribution in the exchange energy, hinting at ideas about new types of density functionals, dedicated to the specifics of the electronic structure of atoms, exploiting the intrinsic spherical symmetry.

## 1. Introduction

The electronic structure of the atoms is tacitly perceived nowadays as something definitively resolved. Aiming at immediate applications, from the start of the era of low power computers, the quantum chemists leaned toward rather drastic approximations and numerical compromises. This trend is continued even nowadays, in the quest for computing larger structures, challenging the domain of nano-chemistry. In this race, feedback with a sound account of the atomic structure is somewhat ignored.

A clear example is the extensive and intensive use of Gaussian type orbitals (GTOs) [1,2]. The users are aware that the *r*^k^·exp(−ζ·*r*^2^) primitives are not the best choices, since the known exact solutions for the hydrogen atom suggests that the Slater type orbitals (STOs) [3], with *r*^k^·exp(−ζ·*r*) components, are more natural options. The GTO-based codes [4,5] are more massively employed than the rather rare STO-type ones [6]. In addition, as we observed recently [7], the GTO computational establishment carries, since early editions of computer codes, a hidden drawback, overlooked by most users. Namely, the GTO codes and repositories are built on drastically limited radial power-patterns of *r*^k^·exp(−ζ·*r*^2^) primitives. Thus, the *s*-type orbitals are always without radial prefactors (i.e., *k* = 0), the *p*-type ones get only *k* = 1, while the *d* functions correspond to *k* = 2, and so on. An inspiration borrowed from hydrogen prototypic exact solution will suggest that a larger variety of *k* powers for each type of shell is conceptually welcomed and technically beneficial. Another collateral loss induced by the success of GTO technology comes from the evaluation of all the integrals, including the atomic ones, by products dichotomized with respect to *x*, *y*, and *z* Cartesian coordinates, because this is technically convenient [8].

However, in this way, a whole wisdom of factorizing the atomic problems in radial and angular parts is lost. The black-box treatment of the atomic integral ignores the advantage of thinking of the inter-electronic effects (Coulomb, exchange, and correlation) with the help of the so-called Slater–Condon integrals [9]:

(1) $$F_{ab}^{k}\equiv F_{n_{a}l_{a},n_{b}l_{b}}^{k}=\int_{r_{1}=0}^{\infty }\int_{r_{2}=0}^{\infty }R_{n_{a}l_{a}}{\left(r_{1}\right)}^{2}R_{n_{b}l_{b}}{\left(r_{2}\right)}^{2}\frac{min{\left(r_{1},r_{2}\right)}^{k}}{max{\left(r_{1},r_{2}\right)}^{k+1}}r_{1}^{2}r_{2}^{2}dr_{1}dr_{2}$$

(2) $$G_{ab}^{k}\equiv G_{n_{a}l_{a},n_{b}l_{b}}^{k}=\int_{r_{1}=0}^{\infty }\int_{r_{2}=0}^{\infty }R_{n_{a}l_{a}}\left(r_{1}\right)R_{n_{b}l_{b}}\left(r_{1}\right)R_{n_{a}l_{a}}\left(r_{2}\right)R_{n_{b}l_{b}}\left(r_{2}\right)\frac{min{\left(r_{1},r_{2}\right)}^{k}}{max{\left(r_{1},r_{2}\right)}^{k+1}}r_{1}^{2}r_{2}^{2}dr_{1}dr_{2}$$

defined as a function of the radial components *R*(*r*) of atomic functions belonging to a given couple of shells, *a* and *b*, each identified with their main and secondary quantum numbers, *n* and *l*. In the body of this article we will perform analyses of the exchange density in atoms, rationalizing the energy with the help of Slater–Condon formulas.

The exchange energy in atoms deserves continuous attention. The importance of which is weighted by the high popularity gained by the application of density functional theory (DFT) in practical calculations [10]. The sensible issue is that practically the whole applied DFT stays on extrapolations originating from the uniform electron gas [11]. The many alleviations of this rather limiting hypothesis, belonging to the classes of generalized gradient approximation (GGA) [12,13] or hybrid functionals [14,15], are tacitly an offshoot of the local density approximation. In the atom, the variation of radial density is very steep, decaying exponentially from the height of the nuclear cusp. In such circumstances, the foundation based on electron gas can be questioned, aiming for new paradigms, and exploiting the regularities due to the spherical symmetry. We will attempt this idea in the actual work.

## 2. Results and Discussion

### 2.1. The Energy of the Atom with a General Shell Occupation Scheme

As a basic tool of investigation in the aimed area, we need an explicit formula, with the function of arbitrary occupations of atomic shells, compatible with calculations performed by state-averaged complete active space (CAS), or, in certain cases, in the restricted Hartree–Fock (RHF) frame. Basic textbooks [16] offer many details of the atomic structure theory, which, however, are not handy and general enough to be applied as ancillary tools, compliant with the modern numeric computational procedures. Then, we will tailor a closed formula for the total atomic energy as a function of shell occupations.

The derivation can be started with master formulas met in the so-called DFT + U methods [17,18], where the two-electron energy of a given configuration, defined by its α and β occupation numbers, is written as:

(3) $$V_{ee}[{\left\{n\right\}}_{\alpha },{\left\{n\right\}}_{\beta }]=\frac{1}{2}\overset{a\in \alpha }{\underset{a}{\sum }}\overset{a\in \alpha }{\underset{a\prime \neq a}{\sum }}n_{a}n_{a\prime }\left(U_{aa\prime }\;-\;J_{aa\prime }\right)\;+\;\frac{1}{2}\overset{b\in \beta }{\underset{b}{\sum }}\overset{b\prime \in \beta }{\underset{b\prime \neq b}{\sum }}n_{b}n_{b\prime }\left(U_{bb\prime }\;-\;J_{bb\prime \prime }\right)\;+\;\overset{a\in \alpha }{\underset{a}{\sum }}\overset{b\in \beta }{\underset{b}{\sum }}n_{a}n_{b}U_{ab}$$

where *U* refers to Coulomb-type integrals, actually equivalent to the *F_ab_*^0^ Slater–Condon parameters, and *J* to the averaged exchange integrals, taken as combinations of *F^k^* or *G^k^* integrals.

The above formulation, ascribed in the spin-unrestricted spirit, does not offer the final answer, being valid for a given *S_z_* = *S* spin projection resulted from the balance of α and β populations, but not for the *S* spin itself. Rethinking the situation as a spin-restricted case, the electron–electron potential for a definite *S* spin state can be obtained as the difference between the above generic formula taken at *S_z_ = S* and those for the *S_z_* = *S* − 1 projection, averaging over the whole set of configurations that span these *S_z_* quantum numbers.

The obtained general formula is given in Equation (4), admitting general occupations, $p_{l_{i}}$, over the whole set of shells, *l* denoting the secondary quantum number, while *i* counts the repetition of the given shell type:

(4) $$E=\underset{l}{\sum }\underset{i}{\sum }p_{l_{i}}h_{l_{i}}+\;\underset{l}{\sum }\underset{i}{\sum }\left\{\frac{1}{2}p_{l_{i}}\left(p_{l_{i}}\;-\;1\right)F_{l_{i}l_{i}}^{0}\;+\;\left[\alpha_{l}\cdot p_{l_{i}}\;+\;\beta_{\;l}\cdot p_{l_{i}}^{2}\;+\;\gamma_{l}\cdot \sigma_{l_{i}}\left(\sigma_{l_{i}}\;+\;1\right)\right]J_{l_{i}l_{i}\;}\right\}\;\;+\;\underset{l\leq l\prime }{\sum }\underset{l_{i}\neq l\prime_{i\prime }}{\sum }\left\{p_{l_{i}}p_{l\prime_{i\prime }}\left(F_{l_{i}l\prime_{i\prime }}^{0}\;-\;\frac{1}{2}J_{l_{i}l\prime_{i\prime }}\right)\;-\;2\sigma_{l_{i}}\sigma_{l\prime_{i^{\prime }}}\cdot J_{l_{i}l\prime_{i\prime }}\right\}$$

The $h_{l_{i}}$ elements are the orbital one-electron energies (kinetic and electron-nuclear parts), the $F_{l_{i}l_{i}}^{0}$ and $F_{l_{i}l\prime_{i\prime }}^{0}$ are the respective intra- and inter-shell Coulomb integrals, while the $J_{l_{i}l_{i}}$ and $J_{l_{i}l\prime_{i\prime }}$ are the corresponding averaged exchange integrals. For a *l^n^* sub-configuration, having $n\;\equiv \;p_{l_{i}}$ electrons in the *l_i_* shell, more exactly with *n*_α_ spin-up and *n*_β_ spin-down particles, the $\sigma_{l_{i}}$ = (*n*_α_ − *n*_β_)/2 denotes the net spin of the shell. The shell-distributed spin quantities are summed to the total spin of the atom $S\;=\;\sum_{l}\sum_{i}\sigma_{l_{i}}$. Note that, according to the previous discussion, the obtained energy refers to *S* as a good quantum number. The tacit assumption is that the spin from possible multiple open shells of the general configuration is coupled all parallel. This condition induces a certain limitation, but yet the situation is sufficiently general.

The coefficients ascribed to the intra-shell exchange were obtained by induction, after analyzing the results of spherical averaging on the all possible sets of integer occupations of the shells. Their expressions were found as follows:

(5) $$\alpha_{l}=\frac{4l+3}{4\left(l+1\right)}\;,\;\beta_{l}=-\frac{2l+3}{8\left(l+1\right)}\;,\;\gamma_{l}=\frac{2l+1}{2\left(l+1\right)}\;,$$

if *l* > 0. For the *s*-type orbitals, there is no intra-shell exchange (all the above factors being null). Table 1 shows the formulas for the inter-shell exchange integrals. The diagonal contains the same type of shells, but spanning different functions.

The *J_ll_* are the average values of the exchange integrals over all the orbital couples within a given shell. For the respective *p*, *d*, and *f* cases, these quantities are

(6a) $$J_{pp}=\frac{1}{5}F_{pp}^{2}$$

(6b) $$J_{dd}=\frac{1}{14}F_{dd}^{2}\;+\;\frac{1}{14}F_{dd}^{4}$$

(6c) $$J_{ff}=\frac{2}{45}F_{ff}^{2}\;+\;\frac{1}{33}F_{ff}^{4}\;+\;\frac{50}{1287}F_{ff}^{6}$$

as function of Slater–Condon radial parameters.

To the best of our knowledge, a closed formula for the atomic energy of the atomic body, as a function of general shell occupation numbers, is not presented in the specialized literature, particularly in the concern of the intra-shell exchange terms fulfilling the meaning of state-averaged CAS over multiple open-shell configurations. 

We realized our own code for atomic calculations based on GTO primitives and explicit use of Slater–Condon integrals. The Formula (4) was checked to yield the same results with state-averaged or single-configuration ab initio calculations. It must be emphasized that retrieving the ab initio result with the above formula is not trivial. First of all, this validates the treatment. Then, recall the above sentence, that the actual quantum chemistry codes are not working with Slater–Condon parameterization, because of the lost interest in radial-angular factorization. However, equating to spherically adapted proper parameters brings transparency in the causal engine and clears the way for new rationales.

### 2.2. A Methodology for the Decomposition of Two-Electron Terms

Before going to the analysis step, to describe the exchange energy, further adequate tools must be prepared. In this view, we will proceed to the decomposition of two-electron terms, conducting their evaluation on numeric grids. Note that we are able to, and we do the full analytic calculation of two-electron integrals, repeating the procedure in the numeric mode only for detective purposes. The numeric integration is better done with a grid with an exponential spacing of the points. Such an integration scheme, due to Weber et al. [19], uses the following definition for the *m*-th point:

(7) $$r_{m}=\delta r_{0}\frac{exp\left(mh\right)\;-\;1}{exp\left(h\right)\;-\;1}$$

depending on the spacing of the first point with respect of the origin, *δr*_0_, and a scale parameter, *h*. The same grid is used in several programs conducting numeric calculations of the atoms, to prepare pseudo-potentials for plane-wave calculations, as is the case of the ATOMPAW module [20] from the ABINIT suite [21].

Deciding a maximal radial extension of the set, *r_max_*, the number of points results as

(8) $$m_{max}=\mathrm{int}\;\left[\frac{1}{h}\ln\left(1\;+\;r_{max}\frac{exp\left(h\right)\;-\;1}{\delta r_{0}}\right)\right]$$

The square brackets meaning the integer value. Conversely, if a certain number of points over a given radial interval is desired, the *h* parameter must be fitted, correspondingly. The points are associated with a set of weights:

(9) $$w_{m}=h\cdot \delta r_{0}\frac{exp\left(kh\right)}{exp\left(h\right)\;-\;1}$$

so that the numeric integration of a given function *f*(*r*) can be formulated as a weighted sum over the grid:

(10) $$\int_{r=0}^{\infty }f\left(r\right)dr\approx \overset{m_{max}}{\underset{m=1}{\sum }}w_{m}\cdot f\left(r_{m}\right)$$

A double sum of this sort being performed for two-dimensional integration.

Then, to account for the given exchange element, *J_ab_*, the entities to be integrated over the 2D grid are

(11) $$X_{mn}^{ab}=r_{m}^{2}r_{n}^{2}R_{a}\left(r_{m}\right)R_{b}\left(r_{m}\right)R_{a}\left(r_{n}\right)R_{b}\left(r_{n}\right)\;\;\overset{m_{max}}{\underset{m=1}{\sum }}\chi_{ab}^{k}\cdot \frac{min{\left(r_{m},r_{n}\right)}^{k}}{max{\left(r_{m},r_{n}\right)}^{k\;+\;1}}$$

The $\chi_{ab}^{k}$ coefficients formalize the definition of *J_ab_* integrals for a given shell couple, *ab*, as a combination of Slater–Condon parameters with *k* superscript. The $\chi_{ab}^{k}$ values can be picked from Equation (6a,b) and from Table 1. Conventionally, the *m* and *n* indices run on the electrons labeled 1 and 2, respectively, in Equations (1) and (2). One may produce a partial summation, emulating the integration over the electron #2, which corresponds to the one-electron operator in the mean-field treatment of an exchange element:

(12) $$\;Y_{m}^{ab}\approx \overset{n_{max}}{\underset{n=1}{\sum }}X_{mn}^{ab}w_{n}\;\;\to \;\;\;\hat{\;J_{ab}}\left(r_{1}\right)$$

The numeric estimation of the whole exchange coupling integrals can be termed as

(13) $$J_{ab}\approx \overset{m_{max}}{\underset{m=1}{\sum }}w_{m}\cdot \;Y_{m}^{ab}\;=\overset{n_{max}}{\underset{n=1}{\sum }}\overset{m_{max}}{\underset{m=1}{\sum }}w_{m}\cdot w_{n}\cdot X_{mn}^{ab}$$

We probed that, in the calculations described below, the numerically estimated integrals resemble the fourth digit, or better (in atomic units), the analytic ones, working over a 300 point grid, established with the following parameters: *δr*_0_ = 0.001 Bohr, *r_max_* = 20 Bohr, and *h* = 0.02.

### 2.3. The Radial Distribution of the Exchange Energy in Atoms

Now we can attempt the aimed for insight into the atomic structure. For the first check, we will confine the calculations to the B-Ne series, first submitted to CAS or RHF calculations, and afterward reloaded in our codes of analytic atomic calculations, followed by numeric decomposition of the exchange integrals. The RHF refers to the closed-shell case of neon, and to the single-configuration (non-degenerate ground state) of the half-filled *p* shell in nitrogen. All the other situations imply a degenerate orbital term (*P*), treated as CAS average over the corresponding three states.

**Figure 1:**
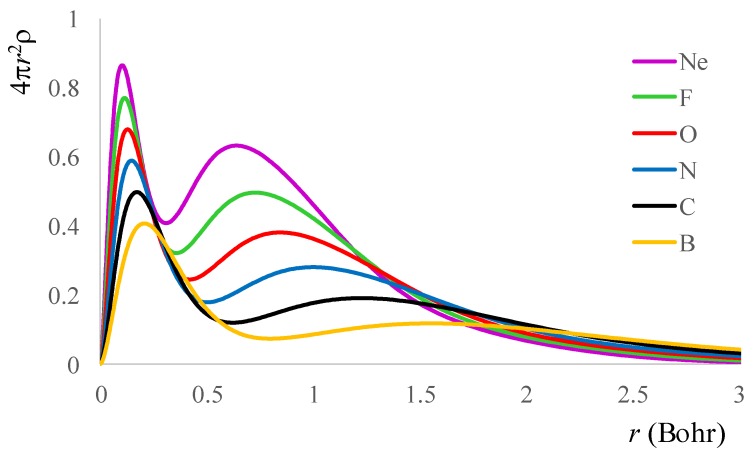
The radial electronic density distribution for the B-Ne atoms series.

Let us start with the simple task of drawing the radial density profiles, as shown in Figure 1. One may note that going from B to Ne, the area under the curves increased, directly corresponding to the total number of electrons in the atoms. The main changes occurred in the part corresponding to the progressive population of the *p* shell.

In the following, we produce the density distribution of the exchange. With the leverage described in Equation (12), one may select from the sum of total energy; in Equation (4), the exchange components integrated only on one electron:

(14a) $$V_{x}^{ll^{\prime \;}}\left(r_{1}\right)=\underset{l\leq l\prime }{\sum }\underset{l_{i}\neq l\prime_{i\prime }}{\sum }\left\{\;-\;p_{l_{i}}p_{l\prime_{i\prime }}\left(\frac{1}{2}\;+\;2\sigma_{l_{i}}\sigma_{l\prime_{i^{\prime }}}\right)\cdot {\hat{\;J\;}}_{l_{i}l\prime_{i\prime }}\left(r_{1}\right)\right\}$$

(14b) $$V_{x}^{l}\left(r_{1}\right)=\underset{l_{i}}{\sum }\left[\alpha_{l}\cdot p_{l_{i}}\;+\;\beta_{\;l}\cdot p_{l_{i}}^{2}\;+\;\gamma_{l}\cdot \sigma_{l_{i}}\left(\sigma_{l_{i}}\;+\;1\right)\right]{\hat{\;J\;}}_{l_{i}l_{i}}\left(r_{1}\right)$$

**Figure 2:**
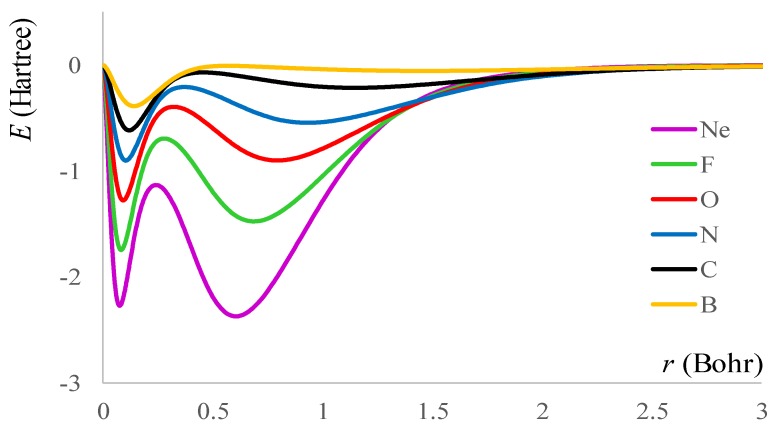
The radial density of the exchange energy in the B-Ne atoms series.

Then, summing over all the intra- and inter-shell contributions, we obtained the energy profiles of total exchange density, *V_x_*, shown in Figure 2.

Contemplating Figure 2, one may see that it looks like a “lake” reflection of the “hills” portrayed in Figure 1. This prompts the idea that the exchange *V_x_*(*r*) ≡ *V_x_*[*ρ*(*r*)] in an atom is better related to the radial density distribution, *r*^2^*ρ*(*r*), than with the *ρ*(*r*)^4/3^ celebrated formula originating from the theory of uniform electron gas.

**Figure 3:**
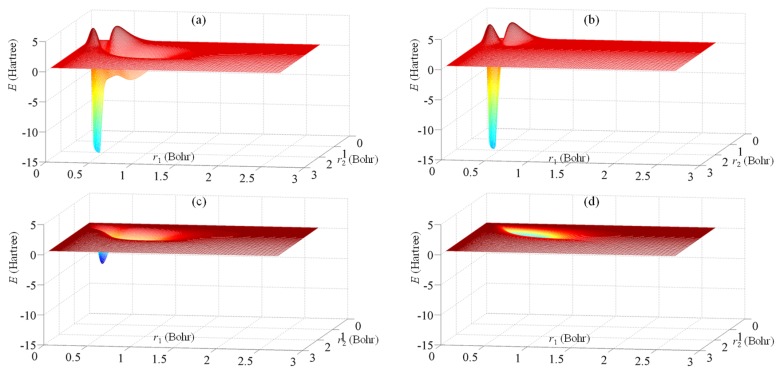
The exchange energy distribution for Ne atom, as function of radial coordinates of formal electrons 1 and 2. (**a**) The total exchange energy; (**b**) the exchange due to inter-shell s-s interaction; (**c**) the s-p exchange coupling; (**d**) the exchange energy inside the *p* shell. The color map goes from blue, at minimum, trough yellow at intermediate negative values, to red for small negative, small positive and null values.

Figure 3 details the exchange energy in the *X_mn_* components derived from Equation (11) for the case of a neon atom. The maps for the other atoms look qualitatively similar. Figure 3a sums all the *X_mn_* contributions at each (*m*,*n*) point of the 2D radial grid. The other panels detail the distinct shell contributions. Figure 3b shows the interaction between the two occupied *s* shells, which can be ascribed as $X_{mn}^{1s,2s}$. This shows positive zones, due to the negative 1s(*r*_1_)2s(*r*_2_) areas, whose action was amended with the negative sign of the exchange in the total energy, −2*J*_1s,2s_. However, the total balance of the exchange was negative due to the net positiveness of the exchange parameters. Figure 3c shows the *s–p* inter-shell exchange, each point containing the $X_{mn}^{1s,2p}\;+\;X_{mn}^{2s,2p}$ terms. Figure 3d shows the *p* intra-shell exchange, small in relative value, as compared to the other parts. For comparability, all the 3D maps from Figure 3 (exchange energy vs. *r*_1_ and *r*_2_ grid points) are drawn at the same vertical scale.

In the following, we will extract a function useful for the discussion of the Fermi hole, *h*(*r*_1_,*r*_2_). Namely, considering the generic formulation of the exchange energy as integration over *ρ*(*r_1_*)*h*(*r*_1_,*r*_2_)/*r*_12_, we emulate *h*(*r*_1_,*r*_2_) multiplying the exchange potential by *r*_12_ and dividing by *ρ*(*r_1_*). The transformation *V_x_r_12_*/*ρ*(*r_1_*) is applied to all the exchange components. Thus, with this reasoning, we propose as interesting quantities the following transformations of Slater–Condon primitives involved in the exchange integrals:

(15) $$u_{ab}\left(r_{1},r_{2}\right)=\frac{1}{\rho \left(r_{1}\right)}R_{n_{a}l_{a}}\left(r_{1}\right)R_{n_{b}l_{b}}\left(r_{1}\right)R_{n_{a}l_{a}}\left(r_{2}\right)R_{n_{b}l_{b}}\left(r_{2}\right)\frac{min{\left(r_{1},r_{2}\right)}^{k}}{max{\left(r_{1},r_{2}\right)}^{k}}r_{2}^{2}$$

**Figure 4:**
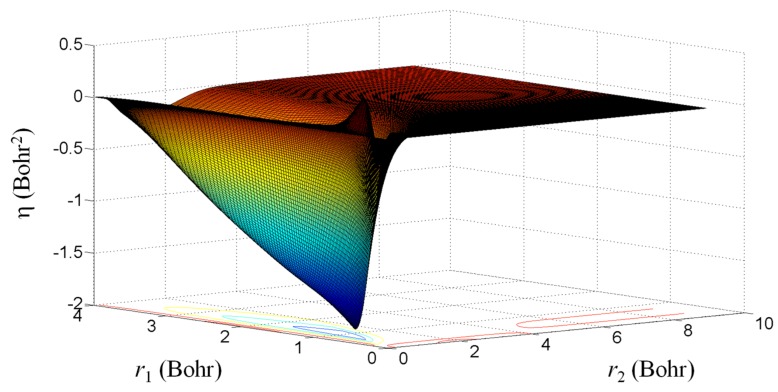
The grid representation of the quantity defined in Equation (15). The color map follows the same convention as in Figure 3.

The above expression is the result after converting the *r*_12_ factor in terms of minimum and maximum from individual electron coordinates, *r*_1_ and *r*_2_, via multipolar expansion, implicit in the definition of Slater–Condon parameters. Performing such a mutation in the body of each exchange term and summing them in the same way as used for the obtaining of the total energy, one draws the map seen in Figure 4. The standard definition of the Fermi hole follows the integration over the elements of density matrices [22,23]. The above procedure can be taken as an alternative way, useful in the further quest for new empirical recipes for the Fermi hole shape. Obeying the demands of spherical symmetry, we propose here the idea of redrawing the Fermi zone in atoms as a spherical crust, i.e., a radial profile, instead of the actual image, as a local hole in the homogenous or non-homogenous electronic density. Analyzing sections along the *r*_2_ coordinate in Figure 4, one may believe that a sharp Gaussian profile of the Fermi radial distribution may be a reasonable approximation. The subject demands further investigation and come-back studies on different classes of atoms and bases.

## 3. Methods

The primary calculations were done with GAMESS (General Atomic and Molecular Electronic Structure System) code [24], using the 6-31+G* basis set [25]. The developed analyses were done with original codes written in the MATLAB–Octave environment [26,27].

## 4. Conclusions

This work takes a constructive critical contribution the actual state-of-the-art in quantum description of the electronic structure in atoms. The actual GTO-based technologies removed the conceptually valuable idea of radial-spherical factorization and the explicit account of the Coulomb, exchange, and correlation effects in the atom by the Slater–Condon parameters. To restore transparency, we derived a handy general formula for the atom in arbitrary shell occupations, obeying the total spin resulted from open shells, as good quantum number. This formula reproduces results from CAS and RHF calculations with dedicated ab initio codes, being implemented with the analytical expansion of all the integrals, in original MATLAB–Octave scripts. In addition, the Slater–Condon parameters were evaluated by numeric quadrature, this way enabling the insight, by analyzing the elementary contributions. In this manner, we drew the radial distribution in the exchange energy density for a prototypic series, including the B-Ne atoms. We found a clear qualitative correlation of the exchange with the radial electronic density distribution. Further numeric handling and corresponding mapping suggested the idea of new density functionals dedicated to the specific of spherical atomic symmetry. Namely, instead of considering the Fermi hole as a local void, it is more appropriate to regard it as a depletion on a spherical crust (i.e., as a radial profile), obeying the spherical symmetry intrinsic to the atom. We will develop this paradigm in future investigations.

## Figures and Tables

**Table 1:** Generic formulas for inter-shell averaged exchange integrals.

Shell Couples	*s*	*p*	*d*	*f*
*s*	$J_{s_{1}s_{2}}=G_{n_{1}sn_{2}}^{0}$			
*p*	$J_{ps}=J_{sp}=\frac{1}{3}G_{sp}^{1}$	$J_{p_{1}p_{2}}=\frac{1}{3}G_{n_{1}pn_{2}p}^{0}\;+\;\frac{2}{15}G_{n_{1}pn_{2}p}^{2}$		
*d*	$J_{ds}=J_{sd}=\frac{1}{5}G_{sd}^{2}$	$J_{dp}=J_{pd}=\frac{2}{15}G_{dp}^{1}\;+\;\frac{3}{35}G_{dp}^{3}$	$J_{d_{1}d_{2}}=\frac{1}{5}G_{n_{1}dn_{2}d}^{0}\;+\;\frac{2}{35}G_{n_{1}dn_{2}d}^{2}\;+\;\frac{2}{35}G_{n_{1}dn_{2}d}^{4}$	
*f*	$J_{fs}=J_{sf}=\frac{1}{7}G_{sf}^{3}$	$J_{fp}=J_{pf}=\frac{3}{35}G_{fp}^{2}\;+\;\frac{4}{63}G_{fp}^{4}$	$J_{fd}=J_{df}=\frac{3}{35}G_{fd}^{1}\;+\;\frac{4}{105}G_{fd}^{3}\;+\;\frac{10}{231}G_{fd}^{5}$	$J_{f_{1}f_{2}}=\frac{1}{5}G_{n_{1}fn_{2}f}^{0}\;+\;\frac{2}{35}G_{n_{1}fn_{2}f}^{2}\;+\;\frac{2}{77}G_{n_{1}fn_{2}f}^{4}\;+\;\frac{100}{3003}G_{n_{1}fn_{2}f}^{6}$
